# Heat impacts on human health in the Western Pacific Region: an umbrella review

**DOI:** 10.1016/j.lanwpc.2023.100952

**Published:** 2023-11-04

**Authors:** Y.T.Eunice Lo, Emily Vosper, Julian P.T. Higgins, Guy Howard

**Affiliations:** a Cabot Institute for the Environment, University of Bristol, UK; b Elizabeth Blackwell Institute for Health Research, University of Bristol, UK; c School of Geographical Sciences, University of Bristol, UK; d Population Health Sciences, Bristol Medical School, University of Bristol, UK; e NIHR Applied Research Collaboration West (ARC West) at University Hospitals Bristol and Weston NHS Foundation Trust, Bristol, UK; f School of Civil, Aerospace and Design Engineering, University of Bristol, UK

**Keywords:** Heat impacts, Mortality, Morbidity, Western Pacific, Climate change

## Abstract

**Background:**

High temperatures and heatwaves are occurring more frequently and lasting longer because of climate change. A synthesis of existing evidence of heat-related health impacts in the Western Pacific Region (WPR) is lacking. This review addresses this gap.

**Methods:**

The Scopus and PubMed databases were searched for reviews about heat impacts on mortality, cardiovascular morbidity, respiratory morbidity, dehydration and heat stroke, adverse birth outcomes, and sleep disturbance. The last search was conducted in February 2023 and only publications written in English were included. Primary studies and reviews that did not include specific WPR data were excluded. Data were extracted from 29 reviews.

**Findings:**

There is strong evidence of heat-related mortality in the WPR, with the evidence concentrating on high-income countries and China. Associations between heat and cardiovascular or respiratory morbidity are not robust. There is evidence of heat-related dehydration and stroke, and preterm and still births in high-income countries in the WPR. Some evidence of sleep disturbance from heat is found for Australia, Japan and China.

**Interpretation:**

Mortality is by far the most studied and robust health outcome of heat. Future research should focus on morbidity, and lower income countries in continental Asia and Pacific Island States, where there is little review-level evidence.

**Funding:**

Funded by the 10.13039/100004423World Health Organization WPR Office.

## Introduction

Average temperatures and extreme heatwaves have increased in Asia,^1^ Pacific Small Islands,^2^ and Australasia^3^ because of human induced climate change. These trends will continue and diverge in the coming decades depending on greenhouse gas emissions. Heat affects human health and wellbeing, and puts a strain on health and care systems globally.^4^

Human exposure to heat can result in mortality through multiple physiological pathways, including increased blood flow to the skin, reduced blood flow to other organs, and cell damage.^5^ It can also increase heart rate, blood viscosity and cholesterol levels, enhancing the risk of cardiovascular dysfunctions such as ischemic stroke and heart disease.^6^^,^^7^ High temperatures and increased sunshine elevate the concentration of particulate matter (PM), surface ozone, sulphate aerosols and other air pollutants,^8^ which can have an inflammatory effect on the airways of susceptible individuals and increase respiratory illnesses.^9^ Longer and earlier pollination seasons in a warming climate increases pollen sensitization rate and allergic symptom duration.^10^

Under conditions of heat stress, increased sweat production can lead to dehydration if water is not adequately replenished in the body. Dehydration and hyperthermia leading to electrolyte and water imbalance place stress on the kidneys.^4^^,^^11^ The impact of heat on dehydration may be increased where individuals engage in moderate to high strenuous activity, such as manual labour in agriculture commonly practiced in low and middle income countries.^12^

When the thermoregulatory capacity of the human body is exceeded, the core body temperature may reach or exceed 40 °C and central nervous systems complications may occur.^13^ This is the definition of a heat stroke, which is a medical emergency. Potential outcomes of a heat stroke include organ dysfunction and death,^4^^,^^13^^,^^14^ and persisting neurological deficits.^13^ For pregnant women who have a compromised ability to thermoregulate because of increased core body temperature and decreased body area-to-mass ratio for heat loss,^15^^,^^16^ fetal development can be at risk.^17^ Preterm birth (birth before 37 weeks of gestation), stillbirth, and low birth weight (live births that weigh below 2500 g) are adverse birth outcomes that have been associated with heat in the literature.^15^

Furthermore, night-time heat can decrease thermal comfort in homes, reducing people's ability to relieve from day-time heat load and disrupting sleep. The urban heat island effect, a phenomenon where urban areas are warmer than surrounding sub-urban or rural areas, is often greater at night, contributing to additional night-time heat to urban inhabitants.^18^

While global^19^^,^^20^ and regional^21^ reports are available in the context of heat and health, no systematic review designed for the World Health Organization (WHO) Western Pacific Region (WPR) exists in the literature. Such a review complements the global overview of evidence contained in the Intergovernmental Panel on Climate Change (IPCC) 6th Assessment Report^20^ by providing more specific indications of adverse health outcomes likely to be found in the WPR, which contains a large and diverse group of countries. Assessing the current state of review level evidence in an umbrella review gives us a great scope to capture the outcomes and geographical diversity in the WPR. It also gives the most consolidated view of evidence, giving great confidence in the findings for informing regional and national decision making and identifying key evidence gaps and research priorities for the WPR and its sub-regions.

This umbrella review is the first consolidated synthesis on heat and health in the WPR and provides a clear indication of the strength of evidence across the region rather in specific countries. Here, we answer the question “How do high daytime and night-time temperatures affect human health in the WPR?” based on published systematic and literature reviews. Six direct heat-related health outcomes are examined: mortality, cardiovascular morbidity, respiratory morbidity, dehydration and heat stroke, adverse birth outcomes, and sleep disturbance.

## Search strategy and selection criteria

This umbrella review adopted methods based on those described in the Cochrane Handbook for Systematic Reviews of Interventions.^22^ Based on the selected health outcomes, search terms were developed for the Scopus and PubMed databases (Supplementary Materials). Searching additional databases was deemed unnecessary because after the Scopus search, only three additional reviews resulted from the PubMed search. Results were limited to systematic reviews, scoping reviews or meta-analyses with full-text available in English. No restrictions were placed on the date of publication. The last search was conducted in February 2023.

Reviews whose title or abstract indicated that they did not include heat as the environmental exposure, reviews that did not associate the selected health outcomes with heat, reviews that were about animals or plants, reviews that focused only on policy or interventions, and reviews that had a geographical focus outside of the WPR, were excluded. After applying the above exclusion criteria to titles and abstracts, full-text screening was carried out. A review was included if it had clear inclusion and exclusion criteria, described its search strategy including search terms, and contained specific information about any of the selected heat-related health outcomes in any of the WPR countries (see Supplementary Materials for detailed eligibility criteria).

Title, abstract and full-text screening were done by two authors (Lo and Vosper) separately. Title and abstract screening were carried out by both authors on search results on the databases. Reviews that passed this stage were downloaded by Lo to Mendeley Reference Manager under six folders, one for each included health outcome, where she then did full-text screening. Vosper used an Excel spreadsheet to manage her list of screened reviews and did full-text screening online. The inter-rater reliability, represented by percentage agreement, was 78% after full text screening. Six conflicts over inclusion arose, and they were resolved in a meeting between the two authors where each review and its justification for inclusion/exclusion was discussed. Specific data for WPR countries were extracted from the included reviews. No additional analysis was performed on the data. We cite the reviews along with the primary studies from which data were extracted in the reference list.

Additional climate change and health reports relevant for the heat-related health outcomes included in this review were included manually. This was based on the lead authors' knowledge of the literature.

With guidance from the WHO WPR Office, regions in the WPR are grouped into (1) high-income countries (HICs), (2) Pacific Island States (PIs), (3) least developed countries and low- and middle-income countries in continental Asia (LDC/LMIC Continental Asia) and (4) upper middle-income countries in continental Asia (UMIC Continental Asia) (Table 1). The quality of evidence for each included heat-related health outcome in each WPR country category was assessed by applying a modified form of the GRADE (Grades of Recommendation, Assessment, Development and Evaluation) tool.^23^ A score of 4 indicates high quality, 3 indicates moderate quality, 2 indicates low quality, and 1 indicates very low quality. The following GRADE assessment process was used. First, a GRADE score of 4 was given. Then, 1 point was deducted for each of these reasons: (i) study limitations (risk of bias), (ii) indirectness of evidence, (iii) inconsistency of results, (iv) imprecision, and (v) publication bias. A GRADE score was not given where no reviews were found for a certain health outcome in a WPR country category.

**Table 1:** Disaggregation of countries or regions within countries based on their geographical and/or economic status.

HICs	Pacific Island states (LDCs, UMICs, HICs)	Continental Asia (LDCs & LMICs)	Continental Asia (UMICs)
Australia Brunei Darussalam Cook Islands French Polynesia Guam Macau Nauru (If remains HIC) New Caledonia New Zealand N Mariana Islands Japan Palau Pitcairn Republic of Korea Singapore	Fiji (UMIC) Kiribati (LDC) Marshall Islands (UMIC) Micronesia (UMIC) Niue (HIC)^a^ Papua New Guinea (LMIC) Solomon Islands (LDC) Samoa (LMIC) Tokelau (LMIC) Tuvalu (LDC) Vanuatu (LMIC) Wallis & Futuna (HIC)^b^	Cambodia (LDC) Lao PDR (LDC) Mongolia (LMIC) Philippines (LMIC) Vietnam (LMIC)	China Malaysia

a Niue is a self-governing territory within the realm of New Zealand with gross domestic product (GDP) per capita and living standards equivalent to a high-income country.

b Wallis & Futuna is a French collectivity and is considered high income.

## Role of funding source

The funder had no role in study design, data extraction or writing of this review.

## Results

**Fig. 1:**
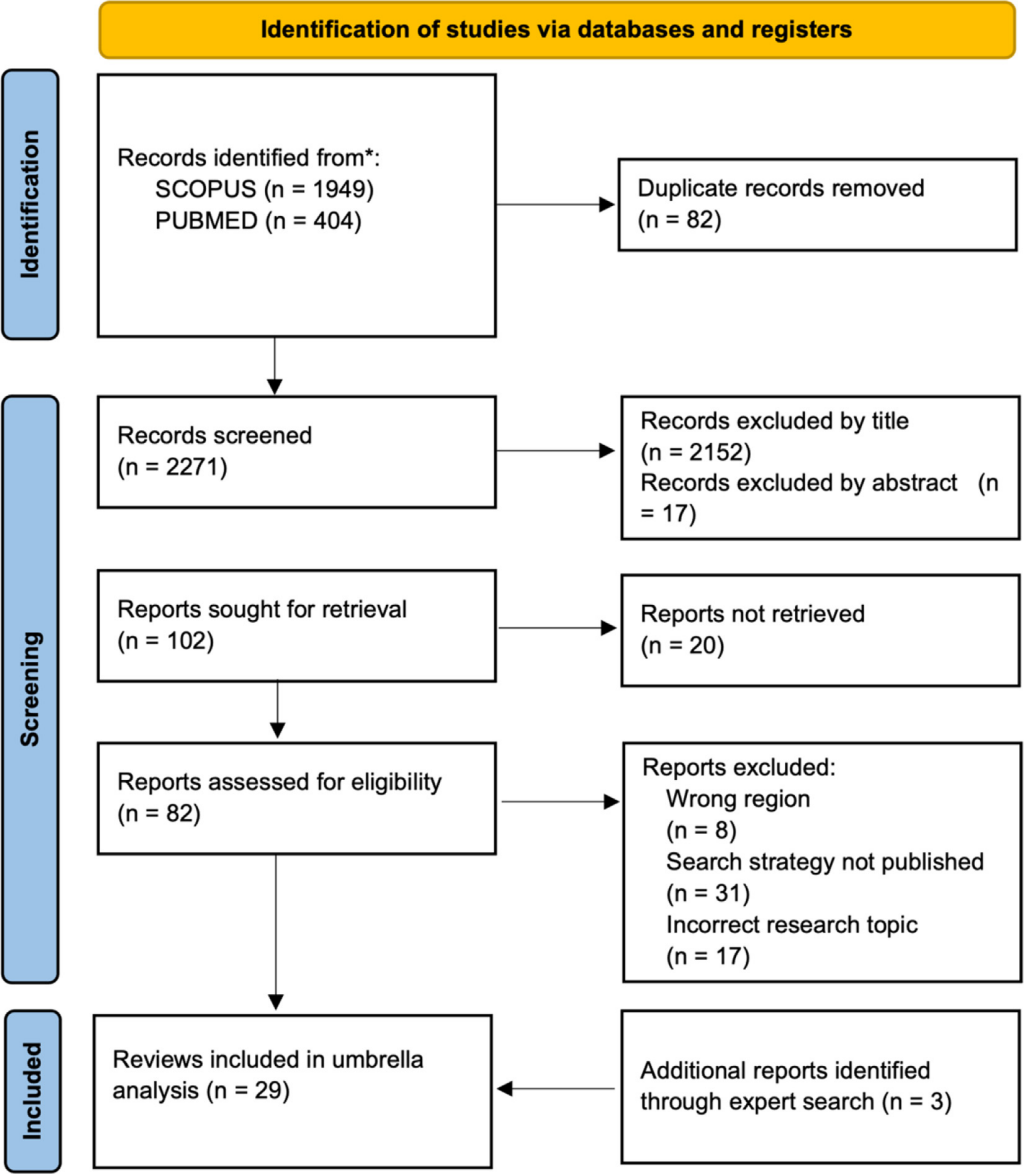
Identification, screening, and inclusion of reviews from database searches, and additional reports identified through authors judgement. The number of excluded reviews and the reason for exclusion are detailed at each stage.

A total of 29 reviews are included in this study (Fig. 1). This consists of 26 reviews resulting from the Scopus and PubMed searches and 3 reports added through authors judgement (Table S1, Supplementary Materials). The three reports are included because they are authoritative climate and health reports relevant for the WPR from the IPCC, Lancet Countdown, and United Nations Children's Fund (UNICEF).

Table 2 provides brief information extracted from each included review or report, collective referred to as ‘included reviews' hereafter for simplicity, grouped by health outcomes. A summary of findings for each health outcome in the WPR country categories is tabulated in Table 3, along with the number of reviews and judgement on their quality. The following sections present the extracted information for each selected health outcome.

**Table 2:** Brief information about the included reviews, including the number of studies therein, their geographical coverage, and the information extracted for specific WPR sub-regions.

Review	Citation	No. of studies	Coverage	Extracted information	Extracted region(s)
**Mortality**					
Campbell et al., 2018	24	188	Global	Older people are more susceptible to extreme heat-related death in Australia and China. Urban heat islands in China increase the risk of impact.	HICs UMIC Continental Asia
Hansen et al., 2011	25	28	Global	Older people with poor health are at a higher risk of heat-related mortality in Australia.	HICs
Mason et al., 2022	26	45	Australia	The most common risk factor for heatwave mortality across Australia is advanced age (above 60 years old).	HICs
Lakhoo et al., 2022	27	26	Global	Increased risk of infant (0–1 year old) mortality per degree increase in temperature.	HICs
Xu et al., 2014	28	12	Global	Larger heatwave mortality in children in Australia and South Korea, compared with other ages. No evidence of this in some other heatwaves in Korea and China.	HICs UMIC Continental Asia
Chan et al., 2019	29	196	China	Stronger heat effect on mortality in urban areas in China. Excess heat-related deaths are associated with cardiovascular and respiratory diseases, and diabetes.	UMIC Continental Asia
Cheng et al., 2019b	30	97	Global	Fractions of all mortality attributable to heat in Australia, Japan, South Korea, and China.	HICs UMIC Continental Asia
Cissé et al., 2022^a^	20	1659	Global	High death tolls and hospitalisations in the 2018 heatwave in Japan.	HICs
Chae and Kim, 2020	31	43	Korea	Numbers of heat-related deaths in South Korea in the 2016 and 2018 heatwaves.	HICs
Luo et al., 2019	32	45	China	Increased rate of non-accidental mortality per degree increase in temperature above threshold.	UMIC Continental Asia
Cai et al., 2021^a^	21	103	China	An increasing trend in heat-related mortality in China, with provinces in east and south-central China being most affected. Heat deaths can be attributed to exacerbation of cardiovascular and respiratory diseases.	UMIC Continental Asia
Ma et al., 2020	33	175	China	Percentage increases in respiratory mortality and chronic obstructive pulmonary disease (COPD) mortality per degree increase in temperature in China.	UMIC Continental Asia
**Cardiovascular morbidity**					
Ma et al., 2020	33	175	China	Positive and significant associations between heat exposure and cardiovascular illnesses in some cities in China.	UMIC Continental Asia
Cheng et al., 2019a	6	54	Global	Non-significant associations between heatwaves and cardiovascular morbidity in Australia, South Korea, and Vietnam. Positive and significant associations for China.	HICs LDC/LMIC Continental Asia UMIC Continental Asia
Phung et al., 2016	30	64	Global	Inconsistent heat-cardiovascular health associations in Australia and Japan. Increased risk of acute myocardial infarction in South Korea and cardiovascular hospital admissions in Vietnam, per degree increase in temperature. Protective effect of heat on ischemic stroke hospitalisation in China.	HICs LDC/LMIC Continental Asia UMIC Continental Asia
Sun et al., 2018	34	30	Global	Increased risk of acute myocardial infarction per degree increase in temperature above a threshold in South Korea.	HICs
Lian et al., 2015	35	20	Global	Protective or non-significant effect of heat on stroke morbidity in China.	UMIC Continental Asia
**Respiratory morbidity**					
Mason et al., 2022	26	45	Australia	Increases in respiratory-related emergency department presentations and ambulance call outs associated with heatwaves in Australia.	HICs
Cheng et al., 2019a	6	54	Global	Non-significant associations between heatwaves and respiratory morbidity in Australia, Vietnam, and China. Positive associations in South Korea.	HICs LDC/LMIC Continental Asia UMIC Continental Asia
Uibel et al., 2022	36	24	High income countries	Excess respiratory-related ambulance call outs in Australia during the 2008 and 2009 heatwaves.	HICs
Grigorieva and Lukyanets, 2021	8	40	Global	Increased risk of acute bronchiolitis-related hospitalisation in young children and respiratory-related emergency visits under high temperatures in China.	UMIC Continental Asia
Hu et al., 2022	37	20	Global	Increased emergency department admissions for childhood asthma due to heat exposure in Australia.	HICs
Xu et al., 2018	38	19	Global	Positive associations between heat and childhood asthma attack in Japan. The risk of asthma hospital admissions increases with temperature in Hong Kong.	HICs UMIC Continental Asia
Bunker et al., 2016	39	121	Global	Non-significant heat effects on respiratory-related hospitalisations and negative effect of heat on asthma-related emergency department visits in South Korea.	HICs
Burton et al., 2011^a^	40	15	Kiribati, Vanuatu	Heat islands in Kiribati and Vanuatu potentially exposing children living in urban areas to heat stress and lung diseases.	PIs
**Dehydration, heat stroke**					
Uibel et al., 2022	36	20	High income countries	Significant increases in renal emergency department presentations and hospital admissions in Australia during heatwaves.	HICs
Li et al., 2015	11	33	Global	Significant increases in renal emergency department presentations and hospital admissions in Australia during heatwaves.	HICs
Nizam et al., 2021	41	20	Global	Male mine workers in Australia show signs of dehydration when working in extreme heat stress.	HICs
**Adverse birth outcomes**					
Ma et al., 2020	33	175	China	Heat exposure increases risk of preterm birth in China.	UMIC Continental Asia
Dalugoda et al., 2022	15	75	Global	High temperatures in the second trimester significantly increase risk of stillbirth in Australia.	HICs
Chersich et al., 2020	16	70	Global	Increased risk of preterm birth in Australia, South Korea and China, and of stillbirth in Australia. No significant association between heat and low birth weight in South Korea.	HICs UMIC Continental Asia
Zhang et al., 2017	42	36	Global	Increased risk of preterm birth in Australia and China. No temperature effect on birth weight in New Zealand.	HICs UMIC Continental Asia
**Sleep disturbance**					
Gulcebi et al., 2021	18	126	Global	Sleep efficiency and rapid eye movement sleep decrease with bedroom temperature in Australia. Standard effective temperature negative impacts sleep quality in China, but not temperature or relative humidity alone.	HICs UMIC Continental Asia
Zisis et al., 2021^b^	43	8	Global	Sleep problems attributed to rising temperatures in Japan.	HICs

The reviews are grouped by the selected heat-related health outcomes.

Some reviews contain relevant information about more than one health outcomes and, therefore, appear more than once in this table. Citations that are not region-specific or are primary research papers cited within the included reviews are excluded from this table.

a Indicates a report included through authors judgement, and its number of studies indicates the total number of citations in that report.

b Indicates an umbrella review, and its number of studies indicates the number of reviews examined in it.

**Table 3:** Summary of findings.

Health outcome	Region	Results	No. of reviews	GRADE
Mortality	HICs	Strong evidence of heat-related mortality in observed warm periods and heatwaves	8	4
	PIs	N/A	0	N/A
	LDC/LMIC Continental Asia	N/A	0	N/A
	UMIC Continental Asia	Evidence of heat-related mortality in China, including cause-specific mortality	7	4
Cardiovascular morbidity	HICs	Inconsistent, weak or no apparent associations between heat exposure and cardiovascular morbidity	3	2
	PIs	N/A	0	N/A
	LDC/LMIC Continental Asia	Non-significant effects of heat on cardiovascular morbidity in Vietnam	2	2
	UMIC Continental Asia	Inconsistent or non-significant associations between heat and cardiovascular health in China	4	2
Respiratory morbidity	HICs	The effect of heat on respiratory morbidity depends on the study location and health indicator	6	2
	PIs	One suggestion of urban heat exposing children in Kiribati and Vanuatu to lung diseases, but without evidence	1	1
	LDC/LMIC Continental Asia	Non-significant associations between heatwaves and respiratory hospital admissions in Vietnam	1	2
	UMIC Continental Asia	Mixed or non-significant evidence of the heat impacts on respiratory health indicators in China	3	2
Dehydration, heat stroke	HICs	Evidence of heat-related dehydration in Australia	3	3
	PIs	N/A	0	N/A
	LDC/LMIC Continental Asia	N/A	0	N/A
	UMIC Continental Asia	N/A	0	N/A
Adverse birth outcomes	HICs	Strong evidence of heat impacts on preterm birth and stillbirth	3	3
	PIs	N/A	0	N/A
	LDC/LMIC Continental Asia	N/A	0	N/A
	UMIC Continental Asia	Evidence of heat impacts of preterm birth in China	3	3
Sleep disturbance	HICs	Some evidence of sleep disturbance from heat in Australia and Japan	2	3
	PIs	N/A	0	N/A
	LDC/LMIC Continental Asia	N/A	0	N/A
	UMIC Continental Asia	Standard effective temperature affects sleep quality in China, but temperature or relative humidity does not	1	2

GRADE stands for Grades of Recommendation, Assessment, Development and Evaluation, and is the adopted approach in this review to assessing the quality of evidence. A GRADE score of 4 indicates high quality, 3 indicates moderate quality, 2 indicates low quality, whereas 1 indicates very low quality. Judgement on (i) study limitations (risk of bias), (ii) indirectness of evidence, (iii) inconsistency of results, (iv) imprecision, and (v) publication bias downgrades GRADE. ‘N/A′ stands for ‘not applicable’ and is used when no review is included for a health outcome in a country category, and therefore no results or GRADE can be given.

For transparency, reviews that are not included in this paper due to meeting exclusion criteria (Search strategy and selection criteria and Fig. 1) are listed in Table S2 in Supplementary Materials.

### Mortality

Everyone can be at risk of heat-related mortality, but some people are more vulnerable than others. Older people are more at risk of heat-related mortality than other age groups in Australia and China.^24^, ^25^, ^26^ The evidence of heat-related child mortality is less consistent for the WPR. Increased risk of infant mortality has been reported in South Korea based on data from seven cities in 2004–2007.^27^^,^^44^ Larger increases in mortality in 0–14 year-olds than other age groups were observed in the 1994 heatwave in South Korea.^28^^,^^45^ However, non-significant changes in mortality in 0–14 year-olds during 1993–2006 heatwaves in Adelaide, Australia^28^^,^^46^; in 0–4 year-olds in the 2003 heatwave in Shanghai, China^28^^,^^47^; or during major heatwaves in 2000–2007 in Seoul, South Korea have been reported.^28^^,^^48^ These discrepancies may have arisen from methodological differences in heat definition, statistical modelling and age stratification, and the characterstics of individual heatwaves.

In the WPR high-income countries, there is consistent evidence of heat-related mortality (including all ages). Up to ∼1% of year-round all-cause or non-accidental mortality has been attributed to heat in Australia, Japan and South Korea, in periods before 2000 and 2000–2010.^30^, ^35^, ^49^, ^50^, ^51^, ^52^ In particular, the 2018 heatwave in Japan resulted in high death tolls and hospitalisations.^20^^,^^53^ The Korean Centers for Disease Control and Prevention reported 17 and 48 heat-related deaths in the 2016 and 2018 summers, respectively.^31^

There are no reviews in the literature covering heat-related mortality in the Pacific Island States, or in LDCs and LMICs in continental Asia. With most countries in these categories being in the Tropics (Table 1), these results are consistent with a review that stated that tropical climates were under-represented in heat and health impact research.^24^

There is a substantial body of research providing evidence of heat-related mortality in China, a UMIC in continental Asia. Overall, there is a 2% increase in non-accidental deaths per 1 °C increase in temperature above the optimal value.^32^ In the period 2000–2018, between 0.6 and 1.3% of all-cause or non-accidental mortality in China has been attributable to heat.^30^^,^^49^^,^^54^ In 2019, 26,800 heat-related deaths occurred.^21^ A four-fold increase in heat-related mortality between the years 1990 and 2019 indicates a rising trend in heat impact in the country.^21^ East and south-central China have seen the most numbers of deaths from heatwaves.^21^ There is also a stronger heat-mortality effect in cities such as Hong Kong and Shanghai,^24^^,^^29^, ^55^, ^56^ although research on the urban heat island effect on mortality is limited for China.^29^

Excess deaths attributable to heat are seen in the exacerbation of cardiovascular and respiratory diseases in China.^21^^,^^29^ In Jinan, every 1 °C increase above a daily mean temperature of 31 °C (threshold above which excess deaths significantly increases) is associated with a 25% increase in respiratory mortality.^33^^,^^57^ In Nanjing, heatwaves are associated with a 48% increase in chronic obstructive pulmonary disease (COPD) mortality and a 32% increase in respiratory mortality.^33^^,^^58^

### Cardiovascular morbidity

While there is evidence of increased cardiovascular mortality in high temperatures, previous analyses have reported inconsistent, weak or no apparent associations between heat exposure and cardiovascular morbidity or hospitalisations,^59^ including in children.^36^

In Brisbane, Australia, the risk of cardiovascular ambulance attendances is significantly higher when maximum temperatures are above 37 °C for at least 2 days (the local definition of a heatwave).^59^^,^^60^ For younger (25–64-year-old) female indigenous people in Australia, the risk of ischemic heart disease at the 95th percentile of maximum temperature is about 32% higher than the risk at a lower temperature (90th percentile), according to data from the period 1992–2011.^59^^,^^61^ However, inconsistent results or non-significant associations have also been found between heat and cardiovascular hospital admissions, ambulance attendances and stroke in Brisbane,^59^^,^^62^^,^^63^ Perth^59^^,^^64^ and Sydney.^6^^,^^59^^,^^65^

In 16 South Korean cities, the risk of emergency visits due to acute myocardial infarction is 1.26 times higher per degree increase in mean temperature above 28.5 °C (inflection point in the temperature-myocardial infarction relationship), according to data from the period 2006–2010.^34^^,^^59^^,^^66^ However, two other studies using data from different sets of cities or provinces in 2003–2008^6^^,^^67^ and 2013–2014^6^^,^^68^ reported no significant heatwave effects on cardiovascular morbidity. These results suggest heterogeneity depending on the location and cardiovascular health indicator.

In Japan, increased risks of emergency admissions for acute coronary syndrome and intracerebral hemorrhage in the Ina area are associated with temperature increases.^59^^,^^69^ However, the risk of out-patient cardiac arrest in older people aged 75 or above decreases when average temperature increases under warm conditions in Osaka, according to data from 1998 to 2007.^59^^,^^70^ Increases in daily maximum temperature have also been reported to decrease hospital emergency transports for hypertension, a precursor for congestive heart failure, in adults above the age of 65 in Tokyo.^59^^,^^71^

There are no reviews in the literature covering heat-related cardiovascular morbidity in the Pacific Island states.

In Hanoi, Vietnam, although a higher risk of cardiovascular hospital admissions is found per degree increase in temperature above 26 °C (temperature of minimum cardiovascular admissions), this result is not significant at the 95% confidence level.^59^^,^^72^ Similar non-significant results have been found for heatwaves in Ho Chi Minh City and other locations in Vietnam.^6^^,^^59^^,^^73^

In China, there is evidence of positive and significant associations between heatwaves and cardiovascular morbidity.^6^^,^^33^ In Shanghai, an 8% increase in cardiovascular hospital admissions has been found to be associated with a heatwave in 2007.^74^ However, in Jinan, exposure to high temperatures could have a protective effect on ischemic stroke hospitalisation, with exposure to 30 °C being associated with a significantly reduced risk of stroke hospital admissions than exposure to 15 °C.^59^^,^^75^^,^^76^

### Respiratory morbidity

In Australia, there is evidence that heatwaves are significantly associated with increases in respiratory-related emergency department presentations^26^^,^^77^^,^^78^ and ambulance call outs.^26^^,^^60^^,^^79^ In Brisbane during the period 2003–2009, the risk of asthma emergency department admissions in children was 1.8 times at 26.5 °C, compared with 24 °C, with male children and children under the age of 4 being most vulnerable.^37^^,^^80^ In Adelaide, 360 and 518 excess ambulance call outs have been estimated for the 15–64 age group during the 2008 and 2009 heatwaves, respectively.^36^^,^^79^ However, unchanged or even reduced respiratory-related emergency department presentations during the heatwaves have been reported in the same study, suggesting that not all respiratory-related health indicators respond to extreme heat in the same way. Indeed, other studies have reported non-significant associations between heatwaves and respiratory morbidity.^6^^,^^81^

In Japan, higher daily average temperature has been found to increase emergency visits for childhood asthma in Tokyo,^38^^,^^82^ and the odds of summer night-time primary care visits due to childhood asthma attack in Himeiji City.^38^^,^^83^ Conversely, a negative, albeit less strong, association has been found with daily maximum temperature in Tokyo in the same study.

In South Korea, there is general evidence of positive associations between heatwaves and respiratory morbidity.^6^^,^^67^^,^^68^ However, studies have also reported non-significant heat effects on hospitalisations for allergic disease, asthma and other respiratory diseases across 8 Korean cities,^39^^,^^67^ and a drop in the risk of asthma-related emergency department visits when daily mean temperature rises above 21–23 °C in Seoul.^39^^,^^84^

According to a UNICEF report for the countries of Kiribati and Vanuatu, the heat island effect exposes children living in urban areas to heat stress and lung diseases from photo-chemical smog.^40^ However, there is not enough data to confirm the existence of urban heat islands in these countries.

In Vietnam, non-significant associations between heatwaves and respiratory hospital admissions have been reported.^6^^,^^73^

In Hong Kong, China, significantly increased risk of acute bronchiolitis-related hospitalisation in young children under the age of 2 has been shown under high ambient and apparent temperatures for the period 2008–2017.^8^^,^^85^ The risk of asthma hospital admissions across all ages also increases with temperature above 27 °C in the hot season (May to October), peaking at 30 °C.^38^^,^^86^ In Guangzhou, interactions of sulphate aerosols and high daily temperatures between 27 and 31 °C have been shown to significantly increase respiratory-related emergency department visits.^8^^,^^87^ However, pooling togehter two studies in China, one of which reported significantly increased respiratory emergency visit risks in hot weather in Beijing^88^ and the other one did not for respiratory hospital admissions in Shanghai,^74^ another review summarised an overall non-significant association between heatwaves and respiratory morbidity in China.^6^

### Dehydration and heat stroke

Male mine workers in Northern Australia who worked in extreme heat stress (i.e., wet bulb globe temperature above 32 °C) had an increased urine specific gravity (a sign of dehydration) during their shift.^41^^,^^89^ Significant increases in renal emergency department presentations and hospital admissions have also been observed during heatwaves in Adelaide,^36^^,^^79^ Brisbane^11^^,^^90^ and Perth.^11^^,^^64^

Diabetic patients are more prone to both dehydration and heat stroke when exposed to hot weather.^91^ The effects of heat on dehydration and heat stroke have not been found in existing systematic reviews for other WPR countries. This is likely to be due to the close relation between dehydration and cardiovascular health, and between heat stroke and death.

### Adverse birth outcomes

In Brisbane, Australia, there is strong evidence of heat impacts on preterm birth and stillbirth. The odds ratio of preterm birth is at least 1.13 (95% confidence interval: 1.03–1.24) for women who are exposed to at least one heatwave after 20 weeks of gestation and before giving birth.^16^^,^^42^^,^^92^ High temperatures in the second trimester^15^^,^^93^ and the last 4 weeks of pregnancy^16^^,^^94^ also significantly increase the risk of stillbirth, with the association being generally more pronounced towards the later stages of pregnancy.^16^ One study for Brisbane reports a 1.46-fold (hazard ratio; 95% CI: 1.09–1.96) increase in stillbirth risk when heatwave exposure occurs in the eighth gestational month.^16^^,^^95^ In New Zealand, no effect of temperature during any trimester on infant birth weight has been found.^42^^,^^96^

In Seoul, South Korea, exposure to heat during pregnancy has been found to significantly increase the risk of preterm birth. Women with low education levels and socioeconomic status are more at risk, with a 1.1-fold (hazard ratio; 95% CI: 1.03–1.17) increase in preterm birth risk per quartile increase in temperature.^16^^,^^97^ However, the same study did not find a significant association between heat and low birth weight.^16^^,^^97^ The literature is less consistent about the heat impact on birth weight than that on preterm or stillbirth.

No reviews that include adverse heat-related birth outcomes in the Pacific Island States and LDCs or LMICs in continental Asia have been found.

In Chinese cities, exposure to extreme heat during different stages of pregnancy increases the risk of preterm birth in hot areas.^16^^,^^98^ In Guangzhou, exposure to 31.9 °C extreme heat in the last 4 weeks of pregnancy has been found to increase preterm birth risk by 10% (95% CI: 2.9–17.6%), relative to 24.4 °C.^33^^,^^42^^,^^99^ Conversely, one study has reported a protective effect of heat on preterm birth in Shenzhen, with the authors suggesting high air conditioning prevalence in the area being a reason.^16^^,^^42^^,^^100^

### Sleep disturbance

Some evidence of heat impacts on sleep has been found for HICs. A study monitoring indoor temperature and the residents’ sleep quality through wristband sensors in Sydney households reports a 1.04 and 1.65% decrease in sleep efficiency and rapid eye movement sleep (both significant at the 5% level), respectively, per degree increase in bedroom temperature.^18^^,^^101^ Similarly, in Japan, primary physicians conclude that a rising burden of sleep problems could be attributed to rising temperatures.^43^, ^102^, ^103^

No reviews covering the topic of heat and sleep have been found for the PIs and LDCs or LMICs in continental Asia.

For UMICs in continental Asia, information has been found for Beijing, China, where the thermal environment is believed to have a significant effect on sleep quality of participants.^18^^,^^104^ However, the same study reports no correlation between ambient temperature or relative humidity and sleep quality.

## Discussion and conclusions

### Summary of findings

By reviewing 29 reviews in the literature, we have found a strong association between heat exposure and mortality in the WPR. This is supported by observations over long time periods as well as individual heatwaves, in various locations, despite different study designs (see Confounding factors). Older people are more vulnerable to heat-related mortality than other age groups. The same cannot be concluded for young children with existing evidence.

Cardiovascular morbidity indicated by hospital admissions, emergency visits and ambulance attendances, has a moderate association with heat. While a significant positive association has been found for some groups, locations, and heatwaves, non-significant or even negative associations have been found in other cases. A similar moderate association between heat exposure and respiratory morbidity has been found in the WPR.

There is a strong association between heat exposure and dehydration and heat stoke. There is also a strong association between heat exposure and the risks of preterm birth and stillbirth. However, a weak to non-existent association has been found with low birth weight. The association between heat exposure and sleep quality in the WPR is moderate.

### Confounding factors

Studying the effects of heat on humans is complex due to the presence of confounders. Of the 29 reviews examined here, 16 had confounders in their study grading criteria or directly assessed study confounders where relevant. Specifically, five of the reviews^26^, ^27^, ^28^^,^^30^^,^^32^ that focused on mortality included confounders in their results, with the most controlled factor being air pollution, and most commonly particulate matter <10 μm in diameter (PM10) and nitrogen dioxide. Humidity was also noted by^26^ as being an important factor in studying extreme heat and mortality, as the presence of high humidity increases heat stress.

Four of the five cardiovascular reviews^6^^,^^34^^,^^59^^,^^75^ evaluated the studies' ability to include confounders as part of their grading criteria. The most common factors controlled for in these studies were seasonal and long-term trends, with days of the week, holidays, air pollution and the lag effect of temperature included in some studies. None of the reviews made comprehensive summaries on how confounders were treated in the reviewed literature.

Four respiratory reviews^8^^,^^37^, ^38^, ^39^ included confounders in their grading schema. The most comprehensive of which was,^8^ who noted that (i) relative humidity is an important factor along with temperature in determining respiratory system reaction to environmental exposures, and (ii) long-term exposure to high temperatures and air pollution had a larger effect on respiratory health than short-term exposure. Therefore, including a lag in study design is important when studying respiratory responses.

One review^11^ looking at dehydration and heat stroke made note of air pollution being explored in some of their reviewed studies. Among the birth outcome reviews, two^15^^,^^42^ assessed confounders within their search criteria. Specifically,^15^ found that in general studies agreed that heat exposure within the last few weeks of pregnancy may be associated with an increased risk of preterm birth and stillbirth. Neither of the sleep disturbance reviews mentioned confounders, highlighting a lack of existing knowledge in this area.

### Future directions

Heat already adversely affects various aspects of human health in the current climate, albeit to different extents. Of the WPR countries, the proportion of heat-related mortality in the period 1991–2008 attributed to human-induced climate change ranges from 21.3% in China to 61.2% in the Philippines.^105^ Even warmer climates in the future, therefore, pose a substantial risk to human health in the WPR. Future projections suggest that Australia, in particular the northern part, is at a high risk of exceeding wet-bulb temperature thresholds for human survival.^24^ South East Asia will also be one of the worst-hit regions in terms of heat-related mortality in all adaptation scenarios.^20^ In China, 25,800–37,800 additional heat-related deaths per year are projected for 51 cities by the period 2014–2060, depending on the climate change scenario.^29^ A global average warming of 2 °C above pre-industrial levels would see tens of thousands more heat-related deaths, including cardiovascular mortality, in China, compared with 1.5 °C global average warming.^20^^,^^21^

Mitigating climate change by reducing greenhouse gas emissions to net-zero is extremely important to avoid worse heat-health impacts. Simultaneously, adapting the WPR populations to rising temperatures is crucial to reducing present-day impacts and avoiding future impacts. Targeted adaptation strategies for individual WPR countries and the vulnerable groups within them (e.g., older people, city residents, indigenous females, children with asthma, manual labour workers, and pregnant women) are effective ways of increasing population resilience to heat.

Successful adaptation strategies in the WPR include urban planning solutions such as increasing the reflectivity of urban surfaces, shown to work in Melbourne, Australia, and combining cooling materials, increased greenery and shading which showed to reduce peak ambient temperatures in Darwin, Australia.^106^ Moreover, studies reviewing early warning systems in Australia and Japan found them to be an effective ‘no-regret’ public health plan.^107^ While individual members of the WPR have engaged in successful adaptation efforts, different countries may have different constraints and adaptation requirements. We suggest that WPR countries that do not have similar adaptation actions pilot these interventions and test their effectiveness in protecting their population. Because the WPR as a whole is lacking in widespread published and actionable heatwave plans, future research reviewing other feasible adaptation methods for this region is recommended.

**Fig. 2:**
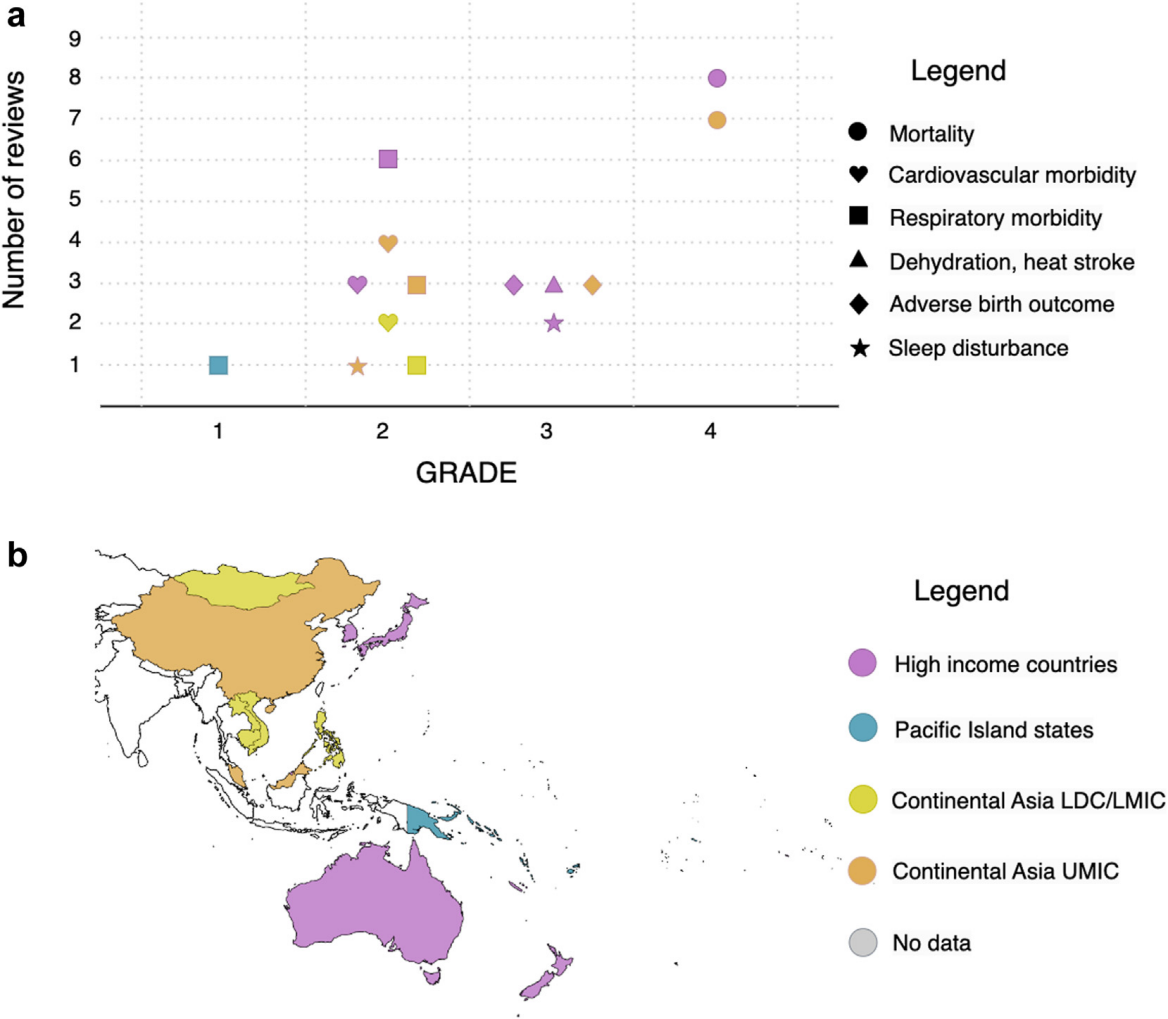
**(a)** The number of extracted reviews and their quality (GRADE score) for the selected health outcomes, which are indicated by the shape of markers. Note that GRADE scores are discrete, with possible values of 1, 2, 3, or 4. Some markers are moved slightly along the x-axis to separate them from other overlapping markers. The colour of the markers indicates country categories for which data have been extracted: high-income countries (pink), Pacific Island States (blue), LDCs or LMICs in continental Asia (yellow), and UMICs in continental Asia (orange). **(b)** A map showing which category (coloured) WPR countries belong to (see also Table 1). American Samoa and Tonga are coloured grey because they are on the WHO WPR country list but not in Table 1. White colour indicates countries that are not on the WHO WPR list.

There are spatial and socio-economic clusters within the WPR on which heat-health research has been focused in the literature. HICs such as Australia, Japan and South Korea have been highly studied, with low to high quality evidence found for all of the included health outcomes (Fig. 2). UMICs in continental Asia, predominantly China, have also been well studied, with low to high quality evidence in all but one included health outcomes (except dehydration, heat stroke). However, only cardiovascular and respiratory morbidity have been systematically reviewed for LDCs and LMICs in continental Asia in the literature, both with low quality (GRADE = 2). The Pacific Island States have hardly been reviewed in terms of heat and health. This could partly be due to the fact that only reviews published in English were examined. Nevertheless, future research on LDCs and LMICs in continental Asia and the Pacific Island States is recommended, as it will help stakeholders better understand the local health risks of heat and ways to reduce them. The compound and cascading health impacts of multiple hazards (e.g., heat and floods) are also important to examine.

This umbrella review has only included reviews published before February 2023, meaning that research published in the most recent 1–2 years would not have been captured. It has also only covered major acute and direct health outcomes of heat, with mortality being the most studied health outcome (Fig. 2). Morbidity is less studied and its association with heat is less robust. Additionally, the casual linkages between heat and injury, the health effects of long-term slow-onset heat exposure, future projections of heat-related maternal, fetal and neonatal health, and the indirect effects of climate change on migration and therefore health and wellbeing are identified research gaps by the IPCC.^20^ Future reviews of primary studies, including the most recent literature, as well as novel primary research in these areas would be highly valuable to provide a fuller picture of the complexity of the heat-health problem in the WPR.

## Contributors

GH developed the scope of this review with WHO's WPR Office. GH and YTEL conceived the review. YTEL developed the search strategy and inclusion/exclusion criteria with input from JPTH and GH. YTEL and EV searched the databases and screened the reviews. YTEL extracted data from the reviews and wrote the manuscript. EV made the figures. JPTH provided guidance on GRADE. All authors edited the manuscript and agreed the final submission.

## Data sharing statement

The sources of all data extracted for this review can be found in References. Search terms used in Scopus and PubMed are in Supplementary Materials.

## Editorial note

The Lancet Group takes a neutral position with respect to territorial claims in published maps and institutional affiliations.

## Declaration of interests

We declare no competing interests.
