# Supplementary material for: Heat impacts on human health in the Western Pacific Region: an umbrella review
Source: Lancet Reg Health West Pac. 2023 Nov 4;42:100952. doi: 10.1016/j.lanwpc.2023.100952 (PMC10652124; doi:10.1016/j.lanwpc.2023.100952)
Supplement: Supplementary material [file mmc1.docx]

**Heat impacts on human health in the Western Pacific Region: an umbrella review**

**Y. T. Eunice Lo^1,2^, Emily Vosper^1,3^, Julian P. T. Higgins^4,5^, Guy Howard^1,6^**

1 Cabot Institute for the Environment, University of Bristol, UK

2 Elizabeth Blackwell Institute for Health Research, University of Bristol, UK

3 School of Geographical Sciences, University of Bristol, UK

4 Population Health Sciences, Bristol Medical School, University of Bristol, UK

5 NIHR Applied Research Collaboration West (ARC West) at University Hospitals Bristol and Weston NHS Foundation Trust, Bristol, UK

6 School of Civil, Aerospace and Design Engineering, University of Bristol, UK

**Supplementary Materials**

**Eligibility criteria:**

- Eligible reviews – reviews that provide their bibliographic search strategy including databases searched and search terms used and present their inclusion and exclusion criteria
- Language: reviews with title, abstract and full text in English were included in the umbrella review
- Date of publication: no restrictions
- Exposure: heat exposure
- Outcome variables: mortality, cardiovascular morbidity, respiratory morbidity, dehydration and heat stroke, adverse birth outcomes, and sleep disturbance
- Associations: reports of statistical associations, qualitatively report associations
- Study location: WPR country (settings of primary studies were reviewed to check for this criterion)
- Eligible reviews that aimed to assess the impact of exposures and outcomes, describe (qualitatively or quantitatively) associations between exposure and outcome variables from a WHO WPR country setting. These associations can be calculated based on a) measured or b) modelled exposure and outcome variables using analytical or numerical models. In the former type of study, the exposure variable must have been measured within a specified period of time prior to sampling or observing the outcome variable. For the latter, modelled values of exposure variables must be based on climate models.

**Database searched:**

Scopus, PubMed

**Data extraction:**

Data were extracted on qualitative and quantitative evidence of associations between the exposure and outcome variables listed above, from countries in the WHO WPR region (https://www.who.int/westernpacific# ). Synthesis done by the reviews was also recorded, including evidence gaps. Results from countries outside of the WHO WPR region were excluded during the title, abstract or full-text stage, when possible.

**Quality assessment:**

The quality of evidence was assessed following the GRADE approach.

**Scopus search terms:**

TITLE-ABS-KEY ( ( ( climate AND change ) OR ( hot AND weather ) OR ( heat ) OR ( heatwave* ) OR ( high AND temperature* ) ) ) AND TITLE-ABS-KEY ( ( ( death* ) OR ( mortality ) OR ( morbidity ) OR ( cardiovascular ) OR ( heat AND stroke ) OR ( asthma ) OR ( respiratory ) OR ( allergy ) OR ( birth ) OR ( lung ) OR ( pneumonia ) OR ( dehydration ) OR ( sleep ) ) ) AND TITLE-ABS-KEY ( ( ( australia ) OR ( brunei ) OR ( cook AND islands ) OR ( french AND polynesia ) OR ( guam ) OR ( hong AND kong ) OR ( macau ) OR ( nauru ) OR ( new AND caledonia ) OR ( new AND zealand ) OR ( northern AND mariana AND islands ) OR ( japan ) OR ( palau ) OR ( pitcairn ) OR ( korea ) OR ( singapore ) OR ( fiji ) OR ( Kiribati ) OR ( Marshall AND Islands ) OR ( Micronesia ) OR ( Niue ) OR ( Papua AND New AND Guinea ) OR ( Solomon AND Islands ) OR ( Samoa ) OR ( Tokelau ) OR ( Tuvalu ) OR ( Vanuatu ) OR ( Wallis AND Futuna) OR ( Cambodia ) OR ( Lao ) OR ( Mongolia ) OR ( Philippines ) OR ( Vietnam ) OR ( China ) OR ( Malaysia ) OR ( west AND pacific ) OR ( global ) ) ) AND TITLE-ABS-KEY ( ( ( review ) OR ( overview ) ) ) AND ( LIMIT-TO ( PUBSTAGE , "final" ) ) AND ( LIMIT-TO ( DOCTYPE , "re" ) OR LIMIT-TO ( DOCTYPE , "ar" ) OR LIMIT-TO ( DOCTYPE , "ch" ) ) AND ( LIMIT-TO ( LANGUAGE , "English" ) )

**PubMed search terms:**

( ( climate AND change ) OR ( hot AND weather ) OR ( heat ) OR ( heatwave* ) OR ( high AND temperature* ) ) ) AND ( ( death* ) OR ( mortality ) OR ( morbidity ) OR ( cardiovascular ) OR ( heat AND stroke ) OR ( asthma ) OR ( respiratory ) OR ( allergy ) OR ( birth ) OR ( lung ) OR ( pneumonia ) OR ( dehydration ) OR ( sleep ) ) ) AND ( ( australia ) OR ( brunei ) OR ( cook AND islands ) OR ( french AND polynesia ) OR ( guam ) OR ( hong AND kong ) OR ( macau ) OR ( nauru ) OR ( new AND caledonia ) OR ( new AND zealand ) OR ( northern AND mariana AND islands ) OR ( japan ) OR ( palau ) OR ( pitcairn ) OR ( korea ) OR ( singapore ) OR ( fiji ) OR ( Kiribati ) OR ( Marshall AND Islands ) OR ( Micronesia ) OR ( Niue ) OR ( Papua AND New AND Guinea ) OR ( Solomon AND Islands ) OR ( Samoa ) OR ( Tokelau ) OR ( Tuvalu ) OR ( Vanuatu ) OR ( Wallis AND Futuna) OR ( Cambodia ) OR ( Lao ) OR ( Mongolia ) OR ( Philippines ) OR ( Vietnam ) OR ( China ) OR ( Malaysia ) OR ( west AND pacific ) OR ( global ) ) AND ( ( review ) OR ( overview ) )

Filters: Full text, Books and Documents, Meta-Analysis, Systematic Review

**Table S1.** Studies that were included in the umbrella review.

| **Review** | **Title** |
| --- | --- |
| *Included via systematic search* | |
| Cheng et al., 2019 | Cardiorespiratory effects of heatwaves: A systematic review and meta-analysis of global epidemiological evidence |
| Bunker et al., 2016 | Effects of Air Temperature on Climate-Sensitive Mortality and Morbidity Outcomes in the Elderly; a Systematic Review and Meta-analysis of Epidemiological Evidence |
| Uibel et al., 2022 | Association of ambient extreme heat with pediatric morbidity: a scoping review |
| Grigorieva et al., 2021 | Combined effect of hot weather and outdoor air pollution on respiratory health: Literature review |
| Ma et al., 2020 | A review of the impact of outdoor and indoor environmental factors on human health in China |
| Chan et al., 2019 | Health impact of climate change in cities of middle-income countries: The case of China |
| Gulcebi et al., 2021 | Climate change and epilepsy: Insights from clinical and basic science studies |
| Mason et al., 2022 | Systematic review of the impact of heatwaves on health service demand in Australia |
| Campbell et al., 2018 | Heatwave and health impact research: A global review |
| Sun et al., 2018 | Effects of ambient temperature on myocardial infarction: A systematic review and meta-analysis |
| Nizam et al., 2021 | A Review of Heat Stress Impact Towards Construction Workers Productivities and Health Based on Several Heat Stress Model |
| Dalugoda et al., 2022 | Effect of Elevated Ambient Temperature on Maternal, Foetal, and Neonatal Outcomes: A Scoping Review |
| Hu et al., 2022 | Evaluation of climate change adaptation measures for childhood asthma: A systematic review of epidemiological evidence |
| Xu et al., 2014 | The impact of heat waves on children's health: A systematic review |
| Hansen et al., 2011 | Older persons and heat-susceptibility: the role of health promotion in a changing climate |
| Cheng et al., 2019 | Impacts of exposure to ambient temperature on burden of disease: a systematic review of epidemiological evidence |
| Chae et al., 2020 | Research trends in agenda-setting for climate change adaptation policy in the public health sector in Korea |
| Lian et al., 2015 | Short-term effect of ambient temperature and the risk of stroke: A systematic review and meta-analysis |
| Li et al., 2015 | Heat waves and morbidity: Current knowledge and further direction-a comprehensive literature review |
| Luo et al., 2019 | ﻿A systematic review and meta-analysis of the association between daily mean temperature and mortality in China |
| Zisis et al., 2015 | Climate change, 24-hour movement behaviors, and health: a mini umbrella review |
| Lakhoo et al., 2022 | The Effect of High and Low Ambient Temperature on Infant Health: A Systematic Review |
| Chersich et al., 2020 | Associations between high temperatures in pregnancy and risk of preterm birth, low birth weight, and stillbirths: Systematic review and meta-analysis |
| Xu et al., 2018 | The association between ambient temperature and childhood asthma: a systematic review |
| Phung et al., 2016 | Ambient temperature and risk of cardiovascular hospitalization: An updated systematic review and meta-analysis |
| Zhang et al., 2017 | Temperature exposure during pregnancy and birth outcomes: An updated systematic review of epidemiological evidence |
| *Included by authors* | |
| Burton et al., 2011 | Climate Change Impacts on Children in the Pacific: Kiribati and Vanuatu |
| Cissé et al., 2022 | ﻿Chapter 7: ﻿Health, Wellbeing and the Changing Structure of Communities. Climate Change 2022: Impacts, Adaptation and Vulnerability. Contribution of Working Group II to the Sixth Assessment Report of the Intergovernmental Panel on Climate Change |
| Cai et al., 2021 | The 2020 China report of the Lancet Countdown on health and climate change |

**Table S2.** Studies not included in umbrella review.

| **Review** | **Title** |
| --- | --- |
| *Incorrect region* | |
| Liu et al., 2022 | Heat exposure and cardiovascular health outcomes: a systematic review and meta-analysis |
| Syed et al., 2022 | Extreme Heat and Pregnancy Outcomes: A Scoping Review of the Epidemiological Evidence |
| Patz et al., 2014 | Climate change: Challenges and opportunities for global health |
| Kuehn et al., 2017 | Heat Exposure and Maternal Health in the Face of Climate Change |
| Dimitrova et al., 2021 | Association between ambient temperature and heat waves with mortality in South Asia: Systematic review and meta-analysis |
| Turner et al., 2012 | Ambient temperature and cardiorespiratory morbidity: a systematic review and meta-analysis |
| Sexton et al., 2021 | Systematic review of ambient temperature exposure during pregnancy and stillbirth: Methods and evidence |
| Alele et al., 2020 | Epidemiology of Exertional Heat Illness in the Military: A Systematic Review of Observational Studies |
| *Search strategy not published / not a systematic review* | |
| Demain, 2018 | Climate Change and the Impact on Respiratory and Allergic Disease: 2018 |
| Singh et al., 2013 | Heat: Not black, not white. It's gray!!! |
| Kravchenko et al., 2013 | Minimization of heatwave morbidity and mortality |
| D’amato et al., 2020 | The effects of climate change on respiratory allergy and asthma induced by pollen and mold allergens |
| D’amato et al., 2015 | Meteorological conditions, climate change, new emerging factors, and asthma and related allergic disorders. A statement of the World Allergy Organization |
| Bunyavanich et al., 2003 | The impact of climate change on child health |
| Takaro et al., 2015 | Climate change and the new normal for cardiorespiratory disease |
| Kenney et al., 2014 | Heat waves aging and human cardiovascular health |
| Chang et al., 2022 | Aging Hearts in a Hotter, More Turbulent World: The Impacts of Climate Change on the Cardiovascular Health of Older Adults |
| Sheffield et al., 2011 | Global climate change and children's health: Threats and strategies for prevention |
| Forsyth et al., 2022 | It's getting hot in here: heat stroke in children and young people for paediatric clinicians |
| Smith et al., 2019 | Pediatric thermoregulation: Considerations in the face of global climate change |
| Anderko et al., 2020 | Climate changes reproductive and children’s health: a review of risks, exposures, and impacts |
| Dermain et al., 2021 | The Impact of Climate Change on the Pollen Allergy and Sporulation of Allergic Fungi |
| D’amato and Cecchi, 2008 | Effects of climate change on environmental factors in respiratory allergic diseases |
| Patz et al., 2005 | Impact of regional climate change on human health |
| Shea et al., 2008 | Climate change and allergic disease |
| O’Neil et al., 2009 | Preventing heat-related morbidity and mortality: New approaches in a changing climate |
| Herbst et al., 2014 | Heat-related deaths in Adelaide, South Australia: Review of the literature and case findings - An Australian perspective |
| Kim and Kim, 2017 | Effect modification of individual- and regional-scale characteristics on heat wave-related mortality rates between 2009 and 2012 in Seoul, South Korea |
| Khalaj et al., 2010 | The health impacts of heat waves in five regions of New South Wales, Australia: A case-only analysis |
| Yoganathan et al., 2001 | Medical aspects of global warming |
| Goggins et al., 2015 | Using Annual Data to Estimate the Public Health Impact of Extreme Temperatures |
| Massazza et al., 2022 | Climate change, trauma and mental health in Italy: a scoping review |
| Mora et al., 2017 | Twenty-seven ways a heat wave can kill you: Deadly heat in the era of climate change |
| Ahdoot et al., 2015 | Global climate change and children's health |
| Du et al., 2013 | Impacts of climate change on human health and adaptation strategies in South China |
| Council on Environmental Health, 2015 | Global climate change and children's health |
| Huang et al., 2011 | Projecting future heat-related mortality under climate change scenarios: a systematic review |
| Arsad et al., 2022 | The Impact of Heatwaves on Mortality and Morbidity and the Associated Vulnerability Factors: A Systematic Review |
| Han et al., 2023 | Asthma triggered by extreme temperatures: From epidemiological evidence to biological plausibility |
| *Incorrect research topic / didn’t discuss exposure and outcome relationship* | |
| Marx et al., 2021 | Heat waves: a hot topic in climate change research |
| Ebi et al., 2021 | Burning embers: Synthesis of the health risks of climate change |
| Moon, 2021 | The effect of the heatwave on the morbidity and mortality of diabetes patients; a meta-analysis for the era of the climate crisis |
| Bartholy et al., 2018 | A brief review of health-related issues occurring in urban areas related to global warming of 1.5°C |
| Di Cicco et al., 2020 | Climate change and childhood respiratory health: A call to action for pediatricians |
| An et al., 2018 | Global warming and obesity: a systematic review |
| Liu et al., 2021 | Is there an association between hot weather and poor mental health outcomes? A systematic review and meta-analysis |
| Liu et al., 2021 | Hot weather as a risk factor for kidney disease outcomes: A systematic review and meta-analysis of epidemiological evidence |
| Lee et al., 2021 | Heat exposure and workers' health: a systematic review |
| Faurie et al., 2022 | Association between high temperature and heatwaves with heat-related illnesses: A systematic review and meta-analysis |
| Xu et al., 2016 | Impact of heatwave on mortality under different heatwave definitions: A systematic review and meta-analysis |
| Lee et al., 2019 | High Temperatures and Kidney Disease Morbidity: A Systematic Review and Meta-analysis |
| Levi et al., 2018 | Impact of climate change on occupational health and productivity: a systematic literature review focusing on workplace heat |
| Cheng et al., 2014 | Impact of diurnal temperature range on human health: a systematic review |
| Xu et al., 2012 | Impact of ambient temperature on children's health: a systematic review |
| Gao et al., 2014 | Impact of ambient humidity on child health: a systematic review |
| Zhang et al., 2020 | The impact of ambient temperature on the incidence of urolithiasis: a systematic review and meta-analysis |
